# Distinguishing functional from structural roles of conserved pore residues during formate translocation by the FocA anion channel

**DOI:** 10.1002/mbo3.1312

**Published:** 2022-08-16

**Authors:** Michelle Kammel, R. Gary Sawers

**Affiliations:** ^1^ Institute for Biology/Microbiology Martin Luther University Halle‐Wittenberg Halle Saale Germany

**Keywords:** anion channel, conserved residues, FNT family, FocA, formate translocation

## Abstract

The formate‐specific anion channel FocA of *Escherichia coli* belongs to the superfamily of homopentameric formate‐nitrite transporters (FNT). Minimally nine amino acid residues are conserved in the formate translocation pore of each protomer of the pentamer, including a histidine (H209) and a threonine (T91), both of which are crucial for bidirectional formate translocation through the pore. Information regarding in vivo functional or structural roles for the other seven conserved residues is limited, or nonexistent. Here, we conducted an amino acid‐exchange analysis of these seven conserved residues. Using an established formate‐responsive *lacZ*‐based assay to monitor changes in intracellular formate levels and anaerobic growth rate due to the inhibitory formate analog hypophosphite, we identified five of the seven residues analyzed to be important for the structural integrity of the pentamer, in particular, two highly conserved asparagine residues, N213 and N262. The remaining two conserved residues, K156 and N172, were essential for formate/hypophosphite translocation. K156 is located on the periplasmic fringe of the pore and aids the attraction of formate to the channel. Here, we show that this residue is also important for formate efflux from the cytoplasm to the periplasm, suggesting a role in formate release from the pore. N172 could be replaced by alanine with retention of low‐level bidirectional anion translocation function; however, exchange for threonine abolished anion translocation. N172 is, therefore, crucial for bidirectional formate translocation, possibly through its interaction with the conserved pore residue, T91.

## Graphical abstract

The FocA anion channel belongs to the homopentameric formate‐nitrite transporter (FNT) family, and each protomer pore has a highly conserved histidine (H209) and threonine (T91) residue essential for formate translocation. This study categorizes seven other conserved residues that line each channel pore; two residues are relevant for formate translocation and five for pore integrity. Two asparagine residues proved to be important for structural stability of the pore, while K156 and N172 were identified to be crucial for bidirectional formate translocation.

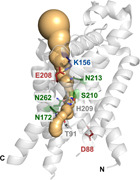

## 1 INTRODUCTION

**Figure 1:**
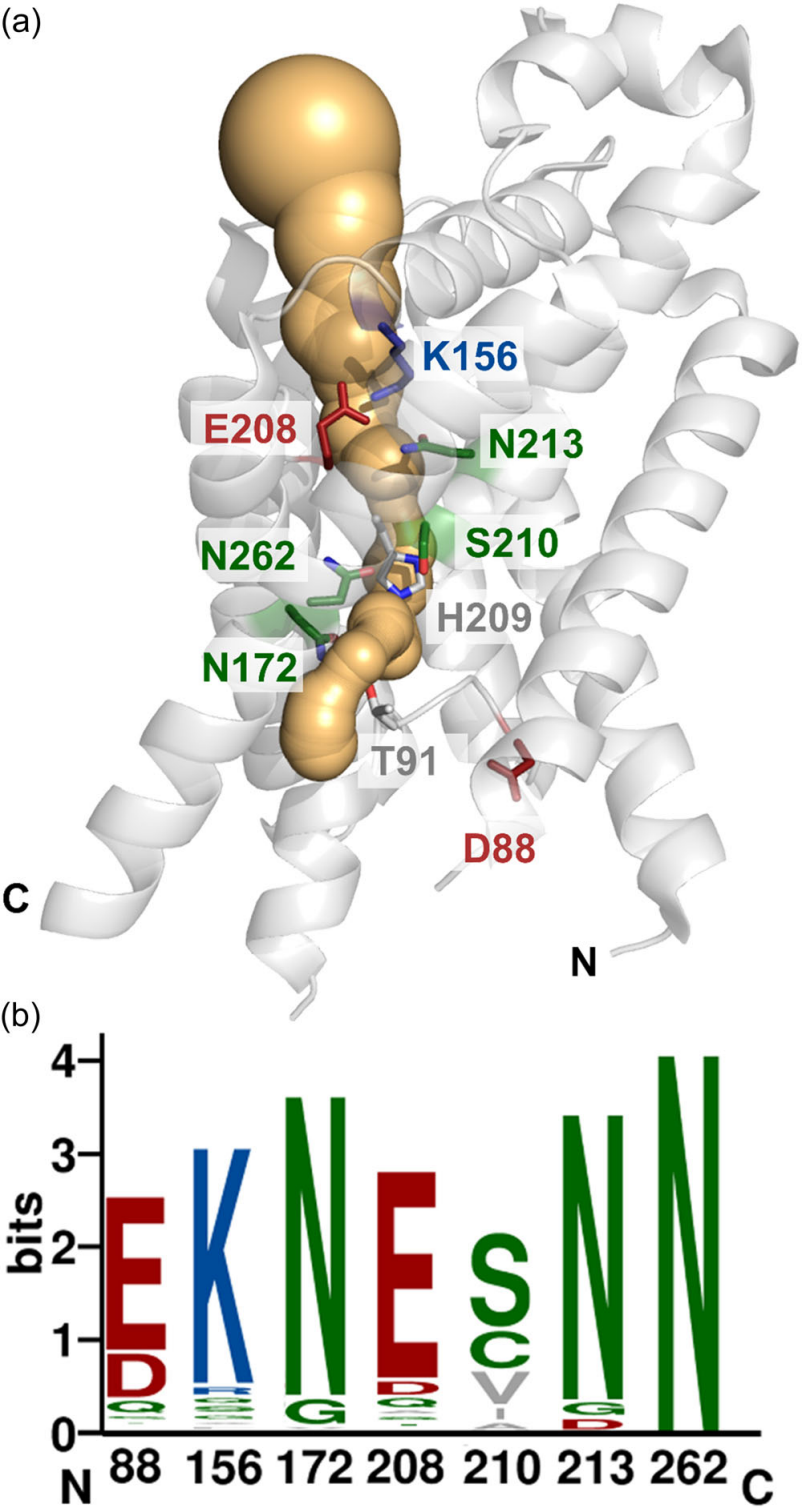
Conserved residues lining the FocA pore. (a) Structural overview of the *Escherichia coli* FocA protomer (Protein Data Bank structure 3KCU; Wang et al., 2009). The peptide backbone of a monomer (gray) is displayed in the cartoon representation and its pore (light orange) was modeled using MOLE 2.5 (see Section 2). The amino acid residues analyzed in this study (b), as well as the mechanistically relevant residues T91 and H209, are shown in the stick representation. (b) Frequency plot of the occurrence of amino acid residues chosen for exchange and subsequent analysis of in vivo anion translocation ability was created using WebLogo 3 (Crooks et al., 2004). A total of 165 annotated formate‐nitrite transporters channels were aligned with native FocA from *E. coli* and assessed with the online tool. “Bits” indicates the relative frequency of occurrence of the residues. The relative percentage conservation of the *E. coli* FocA residues among the 165 formate‐nitrite transporter sequences is as follows: D88 20%, K156 81%, N172 89%, E208 78%, S210 47%, N213 87%, and N262 94%. Amino acid residues with an apolar side chain (A, F, I, L, M, P, V, W) are shown in gray, while polar side chains are displayed in green (C, G, N, Q, T, S, Y). Acidic amino acids are shown in red (D, E) and basic residues are indicated in blue (H, K, R). The coloring of the residues correlates with that used in the structural representation shown in (a).

The homopentameric formate‐nitrite transporter (FNT) superfamily of membrane channel proteins includes several thousand members (Mukherjee et al., 2017). These channels have roles in the translocation of monovalent anions such as formate (represented by FocA), hydrosulfide (HSC), lactate (PfFNT), and nitrite (NirC) (Czyzewski & Wang, 2012; Golldack et al., 2017; Jia & Cole, 2005; Lü, Schwarzer, et al., 2012; Wang et al., 2009). FNTs share strong structural similarities and each protomer of the pentamer exhibits structural homology to the fold of aquaporin channels (Agre et al., 2002; Stroud et al., 2003; Sui et al., 2001). The FNT protomers are tightly packed giving the homopentamer a rigid structure within the membrane (Lü et al., 2013; Waight et al., 2013). Each protomer has a narrow, hydrophobic pore through which the monovalent anionic cargo can pass. Access to the hydrophobic core of the pore is achieved through two funnel‐like vestibules, one on the periplasmic side of the membrane and the other on the cytoplasmic side (Figure 1a). The structural organization of the cytoplasmic vestibule of FocA from *Escherichia coli* is incomplete because the structure of the flexible *N*‐terminal domain of the protein could not be adequately resolved (Wang et al., 2009).

All FNTs have a common set of conserved amino acid residues that line the pore (Figure 1a): these include a centrally located threonine residue (T91 in the numbering of *E. coli* FocA; Figure 1a), which is located on the tip of a flexible Ω‐loop and which links two portions of a broken transmembrane helix (TM2a and b) (Waight et al., 2010) and a histidine residue (H209 in Figure 1a), located on the end of a so‐called S‐loop, which acts as a linker between a second broken transmembrane helix (TM5a and b; Wang et al., 2009). T91 and H209 represent two of the most conserved residues in all FNT channels (Lü et al., 2013; Mukherjee et al., 2017; Waight et al., 2013). An in vivo assay using a formate‐sensitive *fdhF*_*P*_::*lacZ*‐based reporter was established to study FocA from *E. coli* (Beyer et al., 2013; Suppmann & Sawers, 1994). The reporter assay allows monitoring of changes in intracellular formate concentration and has shown that H209 is crucial for pH‐dependent uptake of formate by FocA (Kammel, Trebbin, Pinske, et al., 2022), while T91 has an essential function in formate efflux out of the cell (Hunger et al., 2014; Kammel & Sawers, 2022a, 2022b). X‐ray crystal structure data have revealed that T91 can adopt two positions relative to the fixed location of H209; T91 either forms a hydrogen bond with H209, or it moves roughly 5 Å away from it, toward the cytoplasmic vestibule (Lü, Schwarzer, et al., 2012; Waight et al., 2010). When T91 is separated from H209 it can hydrogen‐bond with asparagine N172, or it can interact with N172 indirectly via a bridging water molecule (Lü, Schwarzer, et al., 2012). Together, these structural observations suggest that N172 might be important for, and involved in, the anion translocation process. N172 belongs to a set of seven other conserved residues lining the pore and for which little or no in vivo information is available regarding any potential functional or structural role each might adopt (Figure 1a,b). The other conserved residues include two further asparagine residues N213 and N262, together with E208, S210, D88, and K156 (Figure 1). Preliminary studies carried out in our lab using the formate‐responsive reporter system showed impaired formate translocation for FocA K156E, E208A, and N213D variants (Hunger et al., 2014). Moreover, heterologous expression studies performed in yeast with K156 amino acid‐exchange variants of FocA from *E. coli* have suggested that it has an important role in the electrostatic attraction of formate to the periplasmic side of the pore, before its pH‐dependent translocation into the cell (Wiechert & Beitz, 2017).

Together, the conservation of T91, H209 plus the seven other residues in the pore strongly suggests that FNTs share a common mechanism to translocate their respective anion cargo. While isolated FNTs are indeed able to translocate several different cargo molecules (Lü, Du, et al., 2012), in vivo data obtained for *E. coli* FocA (Suppmann & Sawers, 1994) indicate that it exhibits specificity for formate, suggesting that other members of the superfamily also might show anion specificity in vivo. In the case of FocA, this anion specificity has been proposed to be achieved through interaction between the cytoplasmic enzyme PflB (pyruvate formate‐lyase) and the *N*‐terminal, cytoplasmically oriented domain of FocA (Doberenz et al., 2014; Kammel et al., 2021). The PflB interaction is suggested to determine the position of T91 (located at the tip of the Ω‐loop) within the pore to control channel gating (Kammel et al., 2021).

The aim of the current study, therefore, is to examine what influence substitutions in the seven conserved residues shown in Figure 1b have on formate translocation out of the cytoplasm. As well as using our formate‐responsive reporter system, we also adopt a hypophosphite‐based growth‐sensitivity assay as a proxy to examine the potential effects of the residue exchanges on formate uptake. Hypophosphite is a chemical analog of formate and, when taken up into the *E. coli* cell by FocA, severely reduces the growth of anaerobically growing *E. coli* cells by irreversibly inhibiting PflB enzyme activity (Suppmann & Sawers, 1994); the reduction in growth rate is due to the prevention of formate and acetyl‐CoA generation from pyruvate (Plaga et al., 1988). Mutants lacking *focA* exhibit resistance to hypophosphite and, consequently, their growth rate is unaffected by low concentrations of the inhibitory anion (Kammel et al., 2021; Suppmann & Sawers, 1994). Using these approaches, we show that the conserved residues fall into two basic categories: those that are required for the structural integrity of the pentamer and consequently the translocation pore; and those that, when exchanged, have a direct impact on anion translocation.

## 2 MATERIALS AND METHODS

### 2.1 Bacterial strains, plasmids, and general cultivation conditions

The strains and plasmids used in this study are listed in Table 1. The cells were grown anaerobically in an M9 minimal medium, pH 7.0 (Sambrook et al., 1989), at 37°C, with 0.8% (w/v) glucose as a carbon source (Kammel et al., 2021). The DH701 (*focA*) strain was transformed with plasmids carrying genes encoding different FocA variants and the cells were cultivated in 15 ml Hungate tubes for studies aimed at determining changes in the intracellular formate concentration using the in vivo *fdhF_P_*::*lacZ* reporter system. To analyze whether the different FocA variants were stably synthesized, growth was carried out in 500 ml serum bottles filled to the top with the medium. In both types of growth, the cells were cultivated statically until they reached the late exponential growth phase (OD_600_ ~0.7−0.9) whereupon they were harvested by centrifugation and analyzed (Kammel et al., 2021).

**Table 1:** Strains and plasmids used in this study

Strains and plasmids	Relevant genotype or characteristics	References or sources	
Strains		
MC4100	F^‐^ *araD* Δ(*argF lac*) *U 169 ptsF25 deoC1 relA1 fblB530 rpsL 150 λ* ^*‐*^	Casadaban (1976)	
DH4100	MC4100 *λ(fdhF::lacZ)*	Hunger et al. (2014)	
DH701	MC4100 *focA* ^*‐*^ *λ(fdhF*_*P*_ *::lacZ)*	Hunger et al. (2014)	
Plasmids			
pfocA (Wt)	Amp^r^, expression vector with the gene *focA* (without Strep II tag)	Kammel et al. (2021)	
pfocA‐D88A	Amp^r^, expression vector with gene *focA* (without Strep II tag), codon aspartate 88 for alanine	This study	
pfocA‐K156A	Amp^r^, expression vector with gene *focA* (without Strep II tag), codon lysine 156 for alanine	This study	
pfocA3‐K156E	Amp^r^, expression vector with gene *focA* carrying a C‐terminal Strep II tag, codon lysine 156 for glutamate	Hunger et al. (2014)	
pfocA‐K156E	Amp^r^, expression vector with gene *focA* (without Strep II tag), codon lysine 156 for glutamate	This study	
pfocA‐N172A	Amp^r^, expression vector with gene *focA* (without Strep II tag), codon asparagine 172 for alanine	This study	
pfocA‐N172T	Amp^r^, expression vector with gene *focA* (without Strep II tag), codon asparagine 172 for threonine	This study	
pfocA‐E208Q	Amp^r^, expression vector with gene *focA* (without Strep II tag), codon glutamate 208 for glutamine	This study	
pfocA‐S210A	Amp^r^, expression vector with gene *focA* (without Strep II tag), codon serine 210 for alanine	This study	
pfocA‐N213A	Amp^r^, expression vector with gene *focA* (without Strep II tag), codon asparagine 213 for alanine	This study	
pfocA3‐N213D	Amp^r^, expression vector with gene *focA* carrying a C‐terminal Strep II tag, codon asparagine 213 for aspartate	Hunger et al. (2014)	
pfocA‐N213D	Amp^r^, expression vector with gene *focA* (without Strep II tag), codon asparagine 213 for aspartate	This study	
pfocA‐N262A	Amp^r^, expression vector with gene *focA* (without Strep II tag), codon asparagine 262 for alanine	This study	
pfocA‐E208Q/N213D	Amp^r^, expression vector with gene *focA* (without Strep II tag), codon glutamate 208 for glutamine and asparagine 213 for aspartate	This study	
pfocA‐N172A/N262A	Amp^r^, expression vector with gene *focA* (without Strep II tag), codon asparagine 172 and 262 for alanine	This study	

The assay to determine the sensitivity of strains toward the toxic formate analog hypophosphite (0.5 mM final concentration) was carried out in microtiter plates, exactly as described (Kammel et al., 2021; Kammel, Trebbin, Pinske, et al., 2022). When required, antibiotics were added to a final concentration of 50 μg/ml for kanamycin and 100 μg/ml for ampicillin.

### 2.2 Construction of plasmids

The expression vector pfocA (Table 1) was used as a template for mutagenesis of the *focA* gene, which in this vector was not fused to codons encoding a *C*‐terminal Strep II‐tag (Kammel et al., 2021). When plasmids carrying genes encoding FocA variants with the *C*‐terminal Strep II‐tag (e.g., pfocA3‐K156E and pfocA3‐N213D; Table 1) were already available, they were used as a template for the introduction of a stop codon at the end of the *focA* gene (using oligonucleotides focA_stop_fw and focA_stop_re, Table A1). Plasmids in which the *focA* gene was mutated at codon 172 (Asn to either Ala or Thr) were generated using nonoverlapping oligonucleotides and the mutagenesis procedure was performed according to the New England Biolabs KLD (Kinase, Ligase, DpnI) mutagenesis protocol (Kammel & Sawers, 2022a). Replacements of the other codons were carried out by site‐directed mutagenesis following the Agilent QuikChange procedure (Kammel, Trebbin, Pinske, et al., 2022).

### 2.3 Analysis of synthesis and membrane integration of FocA variants

The preparation of membrane fractions for analysis of the FocA variants, as well as purification of Strep II‐tagged native FocA, was carried out exactly as described (Kammel et al., 2021; Kammel, Trebbin, Pinske, et al., 2022). The solubilized membrane fractions (50 or 25 μg protein) and 2 µg of purified Strep II‐tagged FocA were separated by denaturing SDS‐gel electrophoresis using 12.5% (w/v) polyacrylamide gels (Laemmli, 1970). As a control for western‐blotting experiments, the polypeptides in the same membrane preparations were separated in a separate sodium dodecyl sulfate polyacrylamide gel electrophoresis [SDS‐PAGE], and afterward, the gel was strained with silver (Kammel et al., 2021; Kammel, Trebbin, Pinske, et al., 2022). Subsequently, immunodetection with anti‐FocA antibodies raised against the full‐length protein (diluted 1:1000) was performed to identify FocA (Kammel et al., 2021; Kammel, Trebbin, Pinske, et al., 2022).

### 2.4 Hypophosphite‐sensitivity test and β‐galactosidase enzyme activity assay

Reduction in the rate of anaerobic cell growth due to supplementation of 0.5 mM sodium hypophosphite to the growth medium was determined as described (Kammel et al., 2021). The intracellular formate level was measured indirectly using the β‐galactosidase enzyme activity assay (Kammel et al., 2021; Miller, 1972).

All analyses were performed in triplicate with minimally three biological replicates and the values obtained are presented with the standard deviation of the mean.

### 2.5 Computational tools

The degree of conservation of the amino acids chosen for substitution was analyzed using the WebLogo tool (online version 2.8.2) (Crooks et al., 2004). The frequency of occurrence of the residues was assessed by analysis of an alignment of 165 annotated FNTs.

A representation of the location of these amino acid residues within the FocA protomer for the translocation pore was visualized with PyMOL (The PyMOL Molecular Graphics System, version 2.5, Schrodinger, LLC). To do this, the pore of either *E. coli* FocA (Protein Data Bank [PDB] structure 3KCU; Wang et al., 2009) or *Vibrio cholerae* FocA (PDB structure 3KLY; Waight et al., 2010) was modeled using the MOLE 2.5 software (Sehnal et al., 2013). The parameters for the calculation of the pore were probe radius 5 Å; interior threshold 1.1 Å; surface cover radius 10 Å; origin radius 5 Å; bottleneck radius 1.2 Å; bottleneck length 3 Å; and cut‐off ratio 0.7. The PyMOL software was also applied for the determination of distances between amino acid residues and for the virtual mutagenesis, in which case different rotamers are shown.

## 3 RESULTS

### 3.1 Conserved amino acid residues lining the FocA protomer pore—Strategy for residue‐exchanges

Apart from T91 and H209, seven other residues line the pore of FNT channels (Figure 1a), which exhibit variable degrees of conservation (Figure 1b). While H209 represents the only charged residue in the core of the pore (Waight et al., 2013), the side‐chain of the neighboring glutamate residue (E208), although oriented away from the pore, is conserved in 78% of the FNTs analyzed (Figure 1b). In FNTs that do not carry glutamate at this position, it is replaced by threonine, glutamine, or aspartate, all of which have hydrogen‐bonding capability. On the other side of H209, there is a serine residue (S210) in *E. coli* FocA, which is less well‐conserved than E208 and its replacements in other FNTs are typically valine, cysteine, isoleucine, or alanine (Figure 1b). Two asparagine residues, N213 and N262, were previously proposed to form key hydrogen‐bonding networks with several residues, including S210 and E208 (Wang et al., 2009). Correspondingly, both residues are well‐conserved with N262 showing 94% conservation (Figure 1b). Together, these four residues are thus all proposed to form an interaction network that stabilizes the position of the S‐loop and H209 (Wang et al., 2009); however, this has never been verified in vivo for these residues.

A further asparagine residue (N172) is located near T91 (Figure 1a) and they form a hydrogen bond when T91 is in the “down” orientation (Wang et al., 2009), or they interact indirectly by coordinating a water molecule between them, which is bound to T91 when in the “up” position in the NirC structure (Lü, Schwarzer, et al., 2012). The findings of these structural studies suggest that N172 might have a key function in anion translocation. The residue is also conserved in almost 90% of FNT channels, with the only naturally occurring variant in some FNTs being a glycine residue (Figure 1b).

At the edge of the periplasmic vestibule, there is a positively charged lysine, K156, which is conserved in approximately 80% of the analyzed FNT proteins (see Figure 1b) and it has been proposed to be important for the uptake of formate by guiding it into the channel through electrostatic attraction (Wiechert & Beitz, 2017). Combined experimental evidence (Hunger et al., 2014; Wiechert & Beitz, 2017), its conservation in numerous FNTs, coupled with the fact that various residues (see Figure 1b) appear to be able to replace it when it is not essential, nonetheless support a function for this residue in most, but not all, channels.

Finally, at the edge of the cytoplasmic vestibule of *E. coli* FocA, there is an aspartic acid residue (D88), while in most other FNT channels, it is replaced by a glutamate residue; consequently, approximately 85% of the 165 analyzed FNT proteins harbor an acidic residue at this position (Figure 1b). Together, these findings suggest that a negatively charged residue might be important at this position.

### 3.2 K156 is essential for formate efflux out of fermenting *E. coli* cells

**Figure 2:**
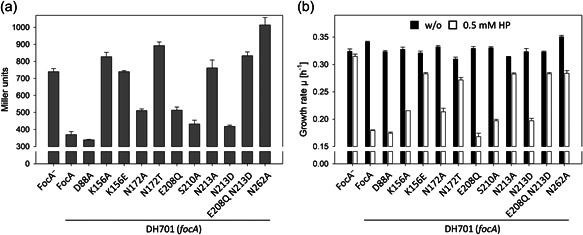
The FocA residues K156, N213, and N262 are essential for bidirectional anion translocation. Formate export and hypophosphite uptake were investigated in the *focA* mutant DH701 transformed with plasmids carrying genes coding for FocA amino acid‐exchange variants. For clarity, the residue exchanged in the respective FocA variants is indicated below each panel. (a) Formate‐induced β‐galactosidase enzyme activity was determined in cells grown to the late‐exponential phase (see Section 2). (b) The anaerobic growth rates of the respective strains were determined after growth in the absence (black histograms) or the presence (white histograms) of 0.5 mM sodium hypophosphite. All experiments were performed with minimally three biological replicates, with each assay carried out in triplicate. The data are presented with the standard deviation of the mean.

Of the seven conserved residues (excepting T91 and H209) lining the FocA pore, exchanges in K156 and E208 have been preliminarily analyzed in vivo using homologous or heterologous hosts (Hunger et al., 2014; Wiechert & Beitz, 2017). While the exchange of K156 for a negatively charged glutamate residue inactivated formate translocation by FocA in an *E. coli* background (Hunger et al., 2014), the exchange of the same residue for either C, A, or M reduced pH‐dependent formate‐uptake activity in a heterologous yeast expression system (Wiechert & Beitz, 2017). In contrast, exchanges in E208 for A or Q had no significant effect on either formate efflux (Hunger et al., 2014) or formate uptake (Wiechert & Beitz, 2017). Here, using our recently optimized in vivo reporter systems to detect changes in formate efflux or hypophosphite uptake (Kammel et al., 2021; Kammel, Trebbin, Pinske, et al., 2022), we re‐examined some of these exchanges as controls for the impact exchanges in the other five conserved residues had on formate translocation and hypophosphite uptake (Figure 2).

The *focA* mutant had a β‐galactosidase enzyme activity of approximately 750 units after anaerobic growth with glucose (Figure 2a). This activity directly reflects formate accumulation inside the cell (Kammel et al., 2021; Kammel & Sawers, 2022b). Introduction of pfocA carrying the gene encoding native FocA resulted in an approximate 50% reduction in formate levels inside the cells, indicating FocA‐dependent formate efflux. In comparison, when E208 was exchanged for glutamine, formate efflux by FocA_E208Q_ was only mildly perturbed and still exhibited roughly 60% of the efflux level (equates with higher β‐galactosidase enzyme activity) compared with native FocA (Figure 2a). The uptake of hypophosphite by this variant was unperturbed and was similar to that determined when the cells synthesized native FocA (Figure 2b). Surprisingly, similar results were observed when alanine replaced E208 (data not shown), indicating that the negatively charged side‐chain of the glutamate residue plays no significant role in forming stabilizing hydrogen bonds under the conditions used in this study and instead, the role of the residue is likely important in stabilizing backbone interactions (Wang et al., 2009).

**Figure 3:**
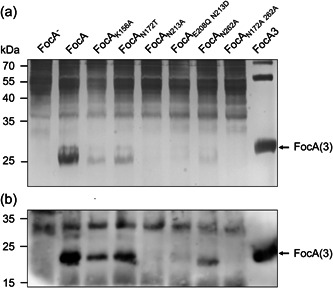
Analysis of synthesis and membrane integration of FocA variants. Samples of membrane fractions (50 or 25 µg protein) and 2 µg of purified Strep II‐tagged FocA were separated in a 12.5% (w/v polyacrylamide) sodium dodecyl sulfate polyacrylamide gel electrophoresis. (a) Silver staining of the separated polypeptides in the membrane fractions (25 µg of protein) derived from strains synthesizing the indicated FocA variants; FocA^‐^ indicates strain DH701 (*focA*). (b) Western blot analysis of polypeptides in membrane fractions (50 µg protein) to detect FocA using anti‐FocA antibodies (1:1000). An aliquot of purified FocA (2 µg) acted as a control. The migration position of FocA (without and with the Strep II‐tag) is indicated on the right‐hand side of each panel, while the positions of the molecular mass markers (PageRuler Prestained Protein Ladder—Thermo Fisher Scientific) are shown in kDa on the left of the gel or blot. The unidentified cross‐reacting polypeptide migrating at approximately 30 kDa acted as a further loading control.

When plasmids carrying mutations in *focA* that resulted in exchanges at K156 were introduced into strain DH701(*focA*), a quite different result was obtained (Figure 2). First, when DH701 synthesized either the FocA_K156A_ or FocA_K156E_ variants, formate levels were similar to those determined for the original *focA* mutant (Figure 2a). This indicates that formate is not exported by these exchange variants. A representative western blot analysis of an isolated membrane fraction from these cells synthesizing FocA_K156A_ revealed that it was stably made and incorporated into the membrane fraction (Figure 3). This finding excludes the possibility that the lack of formate efflux was due to a lack of FocA_K156A_. It should also be noted that increasing the copy number of native FocA by using plasmids, or by increasing gene expression, does not significantly affect the overall kinetics of formate translocation through the growth phase (Beyer et al., 2013; Suppmann & Sawers, 1994). Thus, when a FocA variant fails to translocate formate or hypophosphite, increasing the synthesis level of that variant does not recover translocation ability (Kammel et al., 2021).

Remarkably, the growth of DH701 synthesizing FocA_K156A_ was still highly sensitive to sodium hypophosphite, where an approximate 70% reduction in growth rate was measured compared with when the same strain synthesized native FocA (Figure 2b). This result indicates that hypophosphite uptake by FocA was still functional when an apolar alanine residue replaced the positively charged lysine residue. In contrast, when the K156E exchange in FocA was analyzed, the results revealed a strong reduction in sensitivity to hypophosphite (Figure 2b). This result indicates that the negatively charged glutamate side‐chain likely repelled uptake of the formate analog, and agrees with a previous formate import study using strain DH201 (*focA pflB*) carrying pfocA3‐K156E, where no uptake of formate was detected (Hunger et al., 2014). Together, these results suggest that the lysine residue appears to be required for formate efflux into the periplasm by FocA but it is not essential for hypophosphite import by FocA.

### 3.3 The side‐chains of D88 and S210 are not essential for formate translocation by FocA

When DH701 (*focA*) was transformed with either pfocA‐D88A or pfocA‐S210A and was grown anaerobically in M9‐glucose medium, it had a β‐galactosidase enzyme activity of between 340 and 430 Miller units and consequently had intracellular formate levels similar to the strain synthesizing native FocA (Figure 2a). Similarly, the hypophosphite‐sensitivity profiles of DH701 synthesizing these variants were similar to that of the strain synthesizing native FocA (Figure 2b). These results thus rule out any essential role for the side‐chains of either of these two residues in formate translocation through the FocA pore under these conditions.

### 3.4 Evidence that the conserved glutamine residue N172 has a role in formate translocation

Three conserved glutamine residues line the FocA pore, with N213 and N262 being located in the central regions of the pore. On the other hand, N172 is located in proximity to the essential T91 residue (Figure 1a). Crystal structure data suggest that N172 might be important for anion translocation (Lü et al., 2011; Wang et al., 2009). Consequently, N172 was substituted with either a nonhydrogen‐bonding alanine residue or with threonine, which has a similar size to asparagine and retains hydrogen‐bonding potential. Plasmids carrying the correspondingly modified *focA* genes encoding these protein variants were introduced into DH701 (*focA*) and after anaerobic growth, the changes in the levels of intracellular formate, as reflected by β‐galactosidase enzyme activity, were measured (Figure 2a). The results revealed that, while the FocA_N172T_ variant was severely impaired in formate translocation out of the cell, the FocA_N172A_ variant retained approximately 60% of the translocation ability compared to the native FocA protein. Similarly, when the growth‐phenotype of the respective strains in response to hypophosphite was examined, DH701 synthesizing FocA_N172T_ was nearly insensitive (only 12.3 ± 0.3% growth inhibition, compared to a growth reduction for DH701 of 2.8 ± 0.1%) to the formate analog, while the strain synthesizing FocA_N172A_ retained a significant ability (75%) to take up hypophosphite (Figure 2b). The FocA_N172T_ variant was also stably synthesized and integrated into the cytoplasmic membrane as evidenced by western blot analysis using anti‐FocA antibodies (Figure 3); the near‐wild type translocation capability of FocA_N172A_ obviated the need to analyze protein synthesis by western blot analysis.

### 3.5 Residues N213 and N262 are important for the structural integrity of the FocA pore

Both N213 and N262 are well‐conserved residues in FNTs (Figure 1b) and both are involved in hydrogen‐bond networks that are predicted to stabilize the upper portion of the pore (Waight et al., 2010; Wang et al., 2009). A simple amino acid exchange of either residue for alanine should abolish any possibility of the side‐chain of either residue contributing to the hydrogen‐bond network. Indeed, when a suitably mutated *focA* gene was introduced on pfocA‐N213A or pfocA‐N262A into DH701 (*focA*) to test its influence on intracellular formate levels, both variants failed to show any capability of translocating formate out of the cell (Figure 2a). Similarly, the synthesis of FocA_N262A_ even caused significant intracellular accumulation of formate, possibly also suggesting a role for this residue in the formate translocation process. Western blot analysis of membrane fractions derived from strains synthesizing these variants confirmed that FocA_N262A_ was synthesized, but at significantly lower levels than the native protein (Figure 3b), while FocA_N213A_ could not be detected. The latter finding explains the lack of formate export by the corresponding strain (Figure 2a). Both strains also showed a significant reduction in their capacity to take up hypophosphite (Figure 2b).

To confirm the hypothesis that the asparagine residue at position 213 is important for hydrogen‐bond network stabilization, we also analyzed a variant with an aspartate residue replacing asparagine; the carboxylate group on the side chain of aspartic acid can readily participate in hydrogen‐bond formation. The fact that when strain DH701 synthesized FocA_N213D_ (as previously shown by Hunger et al., 2014), both formate efflux (Figure 2a) as well as hypophosphite‐uptake capability, as exemplified by the reduced anaerobic growth rate of the strain (Figure 2b), were restored to near‐native levels, strongly supports our contention that N213 has primarily a structural role in the maintenance of pore stability. As also suggested for E208, the proposed role of N213 in the structural stabilization of the pore was further supported by combining amino acid exchanges E208Q with N213D, which resulted in a protein that could not be detected in the membrane (Figure 3). The strain was defective in formate efflux and hypophosphite uptake (Figure 2), despite the individual exchanges supporting both translocation functions. Further combinations of residue exchanges (e.g., N172A/N262A) generally yielded unstable FocA variants (Figure 3, and data not shown).

## 4 DISCUSSION

The conservation of several key amino acids that line the pores of the FNT superfamily members strongly suggests that a common mechanism is used for the export and import of anions or their conjugate acids (Mukherjee et al., 2017). While slight variations in the residues present especially in the vestibules of different FNT subfamily members might influence which anion/neutral acid can pass through the respective pore, it has been clearly shown that purified FNT proteins can passage a variety of anions (Lü, Schwarzer, et al., 2012). However, the overall architecture of the pores restricts cargo molecules to small monovalent anions (Lü et al., 2013; Waight et al., 2013). Anion specificity is, therefore, likely determined by specific partner protein(s) that interact with the FNT, as has been proposed for enterobacterial FocA (Doberenz et al., 2014; Kammel et al., 2021). Together, this feature of FNTs makes it all the more important to determine what functions the conserved residues lining the pore have, particularly concerning distinguishing whether they have a structural role or whether they are involved in the translocation process. The functional importance of the conserved histidine (H209) and threonine (T91) residues for formate translocation by FocA of *E. coli* has been established (Kammel, Trebbin, Pinske, et al., 2022; Kammel, Trebbin, Sawers, 2022; Kammel & Sawers, 2022b). Here, we have examined the roles of seven further conserved residues that line the pore of *E. coli* FocA and other FNT members. Our findings reveal that two of these residues (K156 and N172) are important in facilitating formate translocation by FocA, while the remaining five residues (D88, E208, S210, N213, and N262) have more or less important functions in maintaining the integrity of the pore's structure, although it cannot at this stage be excluded that N262 might also be important during anion translocation.

The structurally conserved residues fall into two classes, whereby the two glutamine residues, N262 and N213, have the strongest impact on formate translocation because when they are exchanged for the small apolar residue alanine, they cause severe loss of stability of the respective FocA variants in the membrane. This is in accord with their involvement in stabilizing hydrogen‐bonding networks, as predicted from the crystal structure data (Waight et al., 2010; Wang et al., 2009). They appear to be crucial in maintaining rigidity within the pentamer, particularly in the upper, periplasmically oriented part of the protein (see Figure 1a). In the case of N262, the side chain is located within the hydrogen‐bonding distance of T91‐N172 in the *E. coli* FocA structure when T91 is separated from H209 (Wang et al., 2009). However, in the *V. cholerae* FocA structure, the equivalent Asn residue is at an approximate distance of 2.9 Å from the histidine residue when it forms a hydrogen bond with the threonine (Waight et al., 2010). Thus, N262 might also directly or indirectly influence the ability of H209‐T91 to translocate formate through FocA.

The side‐chains of the other three less well‐conserved residues (D88, E208, and S210) appear to play less important roles in stabilizing the protein because exchanges with residues that have small, compact side‐chains were readily tolerated. It is likely, therefore, that backbone contacts (hydrogen bonds and electrostatic interactions) are more important for these residues (Wang et al., 2009).

These conclusions contrast markedly with the results obtained when residue exchanges in K156 and N172 were undertaken. These data revealed that both have important, but distinct, functions in facilitating formate translocation by FocA. Perhaps the most surprising finding was that when K156 was changed to alanine, FocA was no longer able to export formate from the cytoplasm during exponential growth. Nevertheless, the K156A variant of FocA was still capable of importing hypophosphite. A previous study in which FocA from *E. coli* was synthesized in the heterologous host *Saccharomyces cerevisiae* indicated that exchange of the K156 for either alanine, cysteine, or methionine impaired proton‐coupled uptake of formate into yeast cells (Wiechert & Beitz, 2017). The requirement for low pH was not observed in our study, suggesting that in the natural host, the mechanism of hypophosphite/formate uptake is different than in the heterologous yeast system. The hypophosphite anion has a lower p*K*_a_ (1.1) than that of formate (p*K*_a_ = 3.7), and thus it would be expected that FocA_K156A_ should also take up formate. Indeed, we could also show that when strain DH601, which lacks active PflA and FocA and cannot make formate intracellularly (Kammel et al., 2021), synthesized FocA_K156A_, it was still able to import formate, but FocA_K156E_ was strongly impaired in this regard (data not shown). As has been previously proposed (Kammel, Trebbin, Pinske, et al., 2022), it is likely that additional regulatory features, such as control of formate translocation through interaction with PflB (Kammel et al., 2021), account for the different results determined using the homologous and heterologous systems; yeast cells do not have PflB.

These new results also suggest that K156 appears to be more important for the release of formate into the periplasm than for its reimport into the cell, although the blockage of hypophosphite uptake by generating a K156E exchange variant of FocA indicates that K156 does at least partially contribute to electrostatic attraction of the anion in the uptake direction (Wiechert & Beitz, 2017). Whether K156's involvement in the release of formate is somehow mediated via a long‐range effect induced by the PflB interaction on the cytoplasmic side of the membrane, or via an interaction with a further, as yet unidentified, protein at the periplasmic surface of the membrane, is currently unclear. Providing an answer to this question will require further extensive experimental study.

**Figure 4:**
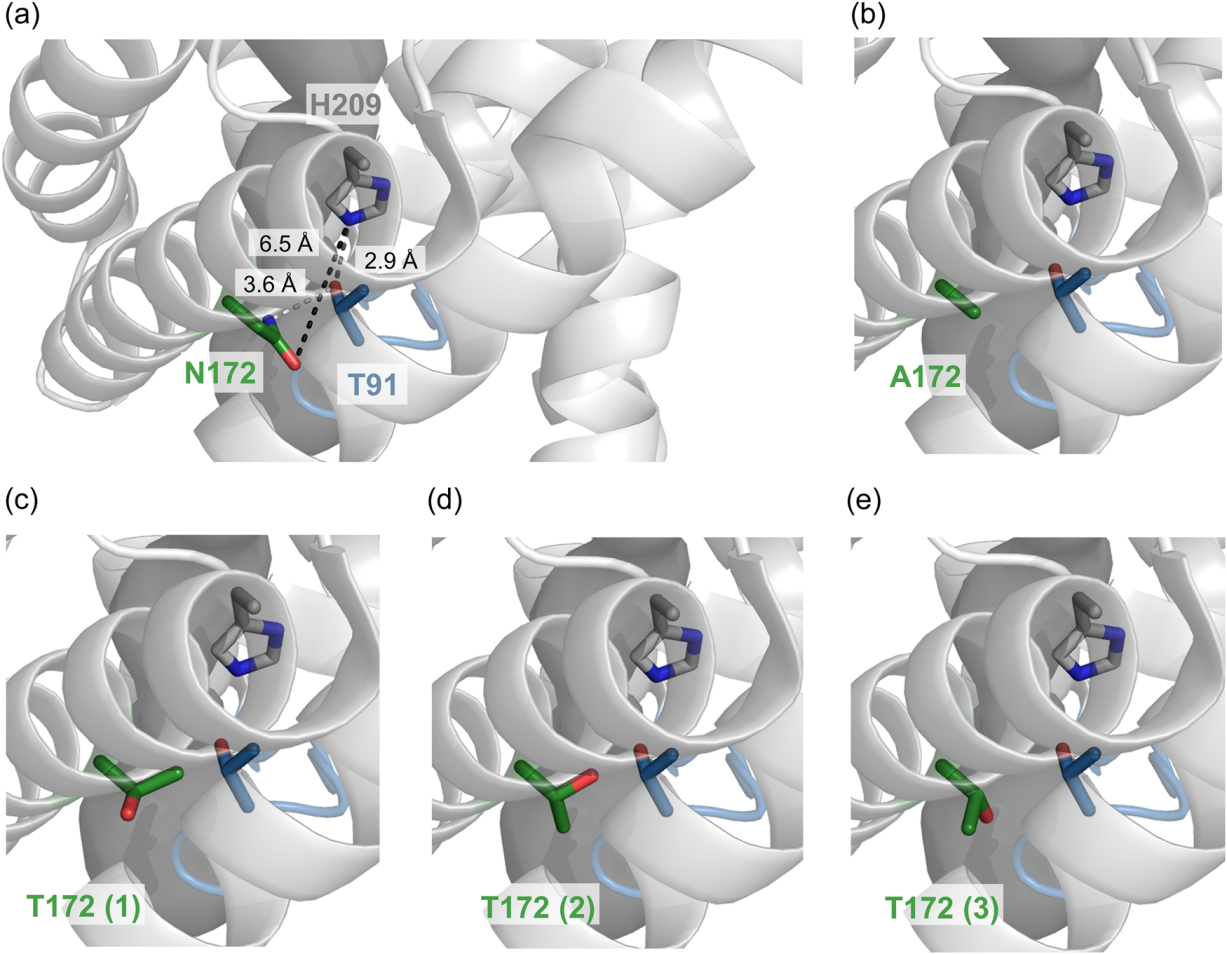
The inactive formate translocation in FocA N172T is due to sterical hindrance within the pore. Structural depiction of the central part of the translocation pore of a *Vibrio cholerae* FocA protomer (Protein Data Bank structure 3KLY; Waight et al., 2010). The peptide backbone of the monomer (chain A) is displayed in cartoon representation in gray and its pore (darker gray) was modeled using MOLE 2.5 (see Section 2). The residues H208 (in gray, corresponding to H209 in *Escherichia coli* FocA), T90 (residue and the Ω‐loop in blue, corresponding to T91 in *E. coli* FocA), and N/A/T171 (in green, corresponding to N172 in *E. coli* FocA) are shown with their respective side chain in stick representation. The distances between the indicated atoms of the side chains were determined using PyMOL (a). PyMOL was also used for the virtual mutagenesis of asparagine N171 to alanine (b) and threonine (c–e). For the threonine exchange, three potential rotamers (T171 1, 2, and 3) are shown and each of them had a predicted frequency of occurrence in this orientation of between 17% and 22%.

The asparagine at residue position 172 is well conserved and the only naturally occurring alternative is glycine, which is found in certain fungi and bacterial FNT proteins of unknown function. Our observation that an exchange of the residue for alanine with its compact methyl side‐chain allowed FocA to retain bidirectional anion translocation function is in accord with a glycine substitution being functional (Figure 1b). The unexpected finding that introduction of a threonine residue prevented both efflux of formate and significantly reduced import of hypophosphite suggests that the γ‐methyl group on its side‐chain sterically blocks the pore (see Figure 4). This would require that the threonine rotamer depicted in Figure 4c (or possibly that shown in Figure 4d) is adopted, thus preventing the passage of formic acid. The alanine residue at position 172 allows clear passage of the acid without imposing any steric hindrance (Figure 4b). The question remains: what selective advantage does asparagine have at this position for formate translocation? Crystal structure data showing an interaction between N172 and T91, either via direct hydrogen‐bond formation (Figure 4a; Lü et al., 2011; Waight et al., 2010) or through joint coordination of a water molecule (Lü, Schwarzer, et al., 2012) observed in the NirC protein, suggests a role for N172 in cargo passage, possibly in the proton‐relay mechanism proposed by Lü et al. (2013). In this proposal, during the uptake of formate, the anion is transiently protonated by H209 to allow passage of the neutral acid through the hydrophobic core of the pore (Atkovska & Hub, 2017). Lü and coworkers (Lü et al., 2013) have hypothesized that after abstraction of a proton from T91 by the imidazolium cation of H209, the now nucleophilic T91 on the tip of the Ω‐loop moves away from the “fixed” H209 residue down toward the cytoplasmic vestibule where recapture of the proton from formic acid occurs. It is proposed that N172 facilitates this process by helping position the nucleophilic T91 residue to recapture the proton. Replacing N172 with alanine resulted in some reduction in hypophosphite translocation capacity, suggesting that the residue exchange reduced the efficiency of translocation, which would be in accord with the proposal above. Further detailed studies will be required to prove or refute this hypothesis, particularly in light of the recent demonstration that uptake of exogenously supplied formate (and possibly hypophosphite) by *E. coli* cells differs mechanistically from the reimport of formate that originated in the cytoplasm and was exported to the periplasm by FocA during exponential growth (Metcalfe et al., 2022).

## 5 CONCLUSIONS

Here, we have been able to characterize an additional seven conserved amino acid residues common to the pores of FNT channels into two categories: those having a structural role; and those with a direct role in formate translocation by FocA. While asparagine residues N213 and N262 likely maintain the integrity of the pore, particularly around the more rigidly structured S‐loop region, three other structurally conserved residues (D88, E208, and S210) tolerate exchange for residues with small or compact side‐chains. This suggests backbone interactions are probably important to aid the structural integrity of the pore in the case of these residues. The stable pentameric structural assembly is likely important for channel function and is similar to what has been observed for the tetrameric aquaporins (Stroud et al., 2003).

A further asparagine residue, N172, was shown to be important in formate translocation through the pore. N172 likely aids correct positioning of, or interaction between, the essential H209 and T91 residues during bidirectional formate/formic acid translocation. Finally, a lysine residue located at the entry to the periplasmic vestibule of FNTs, when exchanged for an apolar alanine residue, proved to be impaired in formate efflux by FocA. This suggests that K156 is required for the release of the anion into the periplasm; the partial retention of hypophosphite uptake by the FocA_K156A_ variant verifies that efflux and uptake of anions by FocA involve different mechanisms.

## AUTHOR CONTRIBUTIONS

**Michelle Kammel**: Conceptualization (supporting); formal analysis (lead); investigation (lead); writing—original draft (supporting); writing—review and editing (equal). **R. Gary Sawers**: Conceptualization (lead); formal analysis (equal); funding acquisition (lead); project administration (lead); supervision (lead); writing—original draft (lead); writing—review and editing (equal).

## CONFLICT OF INTEREST

None declared.

## ETHICS STATEMENT

None required.

## Data Availability

All data generated or analyzed during this study are included in this published article.

## 1

See Table A1.

**Table A1:** Oligonucleotide primers for site‐directed mutagenesis

Primers	Sequence 5′ → 3′^a^
focA_D88A_fw	CTGCGGAGCCGCGCTCTTTACTTC
focA_D88A_rev	GAAGTAAAGAGCGCGGCTCCGCAG
focA_K156A_fw	CAAACCGCCGACCACGCAGTGCACCATACTTT
focA_K156A_rev	AAAGTATGGTGCACTGCGTGGTCGGCGGTTTG
focA_K172A_KLD_fw	TATCCTGGCAGCCCTGATGGTATGTCTG
focA_K172T_KLD_fw	TATCCTGGCAACCCTGATGGTATG
focA_K172_KLD_rev	CCAAGACAGACGGCCTCA
focA_E208Q_fw	GCCAGCGGTTTTCAGCACAGTATCGC
focA_E208Q_rev	GCGATACTGTGCTGAAAACCGCTGGC
focA_S210A_fw	GTTTTGAGCACGCTATCGCAAACATG
focA_S210A_rev	CATGTTTGCGATAGCGTGCTCAAAAC
focA_N213A_fw	CACAGTATCGCAGCCATGTTTATGATC
focA_N213A_rev	GATCATAAACATGGCTGCGATACTGTG
focA_N262A_fw	GTTACGATCGGCGCCATTATCGGTG
focA_N262A_rev	CACCGATAATGGCGCCGATCGTAAC
focA_stop_fw	GAAAACGACCACCATTCAGCTTGGAGCCACCCG
focA_stop_rev	CGGGTGGCTCCAAGCTCAATGGTGGTCGTTTTC

Abbreviation: KLD, Kinase, Ligase and DpnI.

^a^ Underlined bases highlight the substitution introduced.

## References

[mbo31312-bib-0001] Agre, P. , King, L. S. , Yasui, M. , Guggino, W. B. , Ottersen, O. P. , Fujiyoshi, Y. , Engel, A. , & Nielsen, S. (2002). Aquaporin water channels—From atomic structure to clinical medicine. Journal of Physiology, 542(1), 3–16. 10.1113/jphysiol.2002.020818 12096044 PMC2290382

[mbo31312-bib-0002] Atkovska, K. , & Hub, J. S. (2017). Energetics and mechanism of anion permeation across formate‐nitrite transporters. Scientific Reports, 7(1), 12027. 10.1038/s41598-017-11437-0 28931899 PMC5607303

[mbo31312-bib-0003] Beyer, L. , Doberenz, C. , Falke, D. , Hunger, D. , Suppmann, B. , & Sawers, R. G. (2013). Coordinating FocA and pyruvate formate‐lyase synthesis in *Escherichia coli*: Preferential translocation of formate over other mixed‐acid fermentation products. Journal of Bacteriology, 195(7), 1428–1435. 10.1128/JB.02166-12 23335413 PMC3624525

[mbo31312-bib-0004] Casadaban, M. J. (1976). Transposition and fusion of the *lac* genes to selected promoters in *Escherichia coli* using bacteriophage lambda and mu. Journal of Molecular Biology, 104(3), 541–555. 10.1016/0022-2836(76)90120-0 781293

[mbo31312-bib-0005] Crooks, G. E. , Hon, G. , Chandonia, J. M. , & Brenner, S. E. (2004). WebLogo: A sequence logo generator. Genome Research, 14(6), 1188–1190. 10.1101/gr.849004 15173120 PMC419797

[mbo31312-bib-0006] Czyzewski, B. K. , & Wang, D.‐N. (2012). Identification and characterization of a bacterial hydrosulphide ion channel. Nature, 483(7390), 494–497. 10.1038/nature10881 22407320 PMC3711795

[mbo31312-bib-0007] Doberenz, C. , Zorn, M. , Falke, D. , Nannemann, D. , Hunger, D. , Beyer, L. , Ihling, C. H. , Meiler, J. , Sinz, A. , & Sawers, R. G. (2014). Pyruvate formate‐lyase interacts directly with the formate channel FocA to regulate formate translocation. Journal of Molecular Biology, 426(15), 2827–2839. 10.1016/j.jmb.2014.05.023 24887098 PMC5560055

[mbo31312-bib-0008] Golldack, A. , Henke, B. , Bergmann, B. , Wiechert, M. , Erler, H. , Blancke Soares, A. , Spielmann, T. , & Beitz, E. (2017). Substrate‐analogous inhibitors exert antimalarial action by targeting the *Plasmodium* lactate transporter PfFNT at nanomolar scale. PLoS Pathogens, 13(2), e1006172. 10.1371/journal.ppat.1006172 28178358 PMC5298233

[mbo31312-bib-0009] Hunger, D. , Doberenz, C. , & Sawers, R. G. (2014). Identification of key residues in the formate channel FocA that control import and export of formate. Biological Chemistry, 395(7‐8), 813–825. 10.1515/hsz-2014-0154 24659605

[mbo31312-bib-0010] Jia, W. , & Cole, J. A. (2005). Nitrate and nitrite transport in *Escherichia coli* . Biochemical Society Transactions, 33(1), 159–161. 10.1042/BST0330159 15667293

[mbo31312-bib-0011] Kammel, M. , Hunger, D. , & Sawers, R. G. (2021). The soluble cytoplasmic *N*‐terminal domain of the FocA channel gates bidirectional formate translocation. Molecular Microbiology, 115(4), 758–773. 10.1111/mmi.14641 33169422

[mbo31312-bib-0012] Kammel, M. , & Sawers, R. G. (2022a). The autonomous glycyl radical protein GrcA restores activity to inactive full‐length pyruvate formate‐lyase *in vivo* . Journal of Bacteriology, 204(5), e0007022. 10.1128/jb.00070-22 35377165 PMC9112899

[mbo31312-bib-0013] Kammel, M. , & Sawers, R. G. (2022b). The FocA channel functions to maintain intracellular formate homeostasis during *Escherichia coli* fermentation. Microbiology , 168(4). 10.1099/mic.0.001168 35377837

[mbo31312-bib-0014] Kammel, M. , Trebbin, O. , Pinske, C. , & Sawers, R. G. (2022). A single amino acid exchange converts FocA into a unidirectional efflux channel for formate. Microbiology, 168(1), 001132. 10.1099/mic.0.001132 PMC8914244 35084298

[mbo31312-bib-0015] Kammel, M. , Trebbin, O. , & Sawers, R. G. (2022). Interplay between the conserved pore residues Thr‐91 and His‐209 controls formate translocation through the FocA channel. Microbial Physiology, 32, 95–107. 10.1159/000524454 35390794

[mbo31312-bib-0016] Laemmli, U. (1970). Cleavage of structural proteins during the assembly of the head of bacteriophage T4. Nature, 227(5259), 680–685. 10.1038/227680a0 5432063

[mbo31312-bib-0017] Lü, W. , Du, J. , Schwarzer, N. J. , Gerbig‐Smentek, E. , Einsle, O. , & Andrade, S. L. (2012). The formate channel FocA exports the products of mixed‐acid fermentation. Proceedings of the National Academy of Sciences USA, 109(33), 13254–13259. 10.1073/pnas.1204201109 PMC3421167 22847446

[mbo31312-bib-0018] Lü, W. , Du, J. , Schwarzer, N. J. , Wacker, T. , Andrade, S. L. A. , & Einsle, O. (2013). The formate/nitrite transporter family of anion channels. Biological Chemistry, 394(6), 715–727. 10.1515/hsz-2012-0339 23380538

[mbo31312-bib-0019] Lü, W. , Du, J. , Wacker, T. , Gerbig‐Smentek, E. , Andrade, S. L. , & Einsle, O. (2011). pH‐dependent gating in a FocA formate channel. Science, 332(6027), 352–354. 10.1126/science.1199098 21493860

[mbo31312-bib-0020] Lü, W. , Schwarzer, N. J. , Du, J. , Gerbig‐Smentek, E. , Andrade, S. L. A. , & Einsle, O. (2012). Structural and functional characterization of the nitrite channel NirC from *salmonella* typhimurium. Proceedings of the National Academy of Sciences USA, 109(45), 18395–18400. 10.1073/pnas.1210793109 PMC3494889 23090993

[mbo31312-bib-0021] Metcalfe, G. D. , Sargent, F. , & Hippler, M. (2022). Hydrogen production in the presence of oxygen by *Escherichia coli* K‐12. Microbiology , 168(3). 10.1099/mic.0.001167 PMC9558352 35343886

[mbo31312-bib-0022] Miller, J. (1972). Experiments in molecular genetics. Cold Spring Harbor Laboratory.

[mbo31312-bib-0023] Mukherjee, M. , Vajpai, M. , & Sankararamakrishnan, R. (2017). Anion‐selective formate/nitrite transporters: Taxonomic distribution, phylogenetic analysis and subfamily‐specific conservation pattern in prokaryotes. BMC Genomics, 18(1), 560. 10.1186/s12864-017-3947-4 28738779 PMC5525234

[mbo31312-bib-0024] Plaga, W. , Frank, R. , & Knappe, J. (1988). Catalytic‐site mapping of pyruvate formate lyase: Hypophosphite reaction on the acetyl‐enzyme intermediate affords carbon‐phosphorus bond synthesis (1‐hydroxyethylphosphonate). European Journal of Biochemistry, 178(2), 445–450. 10.1111/j.1432-1033.1988.tb14468.x 3061816

[mbo31312-bib-0025] Sambrook, J. , Fritsch, E. F. , & Maniatis, T. (1989). Molecular cloning: A laboratory manual (2nd ed.). Cold Spring Harbor Laboratory.

[mbo31312-bib-0026] Sehnal, D. , Svobodová Vařeková, R. , Berka, K. , Pravda, L. , Navrátilová, V. , Banáš, P. , Ionescu, C. M. , Otyepka, M. , & Koča, J. (2013). MOLE 2.0: Advanced approach for analysis of biomacromolecular channels. Journal of Chemical Information, 5(1), 39. 10.1186/1758-2946-5-39 PMC3765717 23953065

[mbo31312-bib-0027] Stroud, R. M. , Savage, D. , Miercke, L. J. W. , Lee, J. K. , Khademi, S. , & Harries, W. (2003). Selectivity and conductance among the glycerol and water conducting aquaporin family of channels. FEBS Letters, 555(1), 79–84. 10.1016/S0014-5793(03)01195-5 14630323

[mbo31312-bib-0028] Sui, H. , Han, B. G. , Lee, J. K. , Walian, P. , & Jap, B. K. (2001). Structural basis of water‐specific transport through the AQP1 water channel. Nature, 414, 872–878. 10.1038/414872a 11780053

[mbo31312-bib-0029] Suppmann, B. , & Sawers, G. (1994). Isolation and characterisation of hypophosphite‐resistant mutants of *Escherichia coli*: Identification of the FocA protein, encoded by the *pfl* operon, as a putative formate transporter. Molecular Microbiology, 11(5), 965–982. 10.1111/j.1365-2958.1994.tb00375.x 8022272

[mbo31312-bib-0030] Waight, A. B. , Czyzewski, B. K. , & Wang, D.‐N. (2013). Ion selectivity and gating mechanisms of FNT channels. Current Opinion in Structural Biology, 23(4), 499–506. 10.1016/j.sbi.2013.05.007 23773802 PMC3737415

[mbo31312-bib-0031] Waight, A. B. , Love, J. , & Wang, D.‐N. (2010). Structure and mechanism of a pentameric formate channel. Nature Structural and Molecular Biology, 17(1), 31–37. 10.1038/nsmb.1740 PMC3613427 20010838

[mbo31312-bib-0032] Wang, Y. , Huang, Y. , Wang, J. , Cheng, C. , Huang, W. , Lu, P. , Xu, Y.‐N. , Wang, P. , Yan, N. , & Shi, Y. (2009). Structure of the formate transporter FocA reveals a pentameric aquaporin‐like channel. Nature, 462(7272), 467–472. 10.1038/nature08610 19940917

[mbo31312-bib-0033] Wiechert, M. , & Beitz, E. (2017). Mechanism of formate‐nitrite transporters by dielectric shift of substrate acidity. EMBO Journal, 36(7), 949–958. 10.15252/embj.201695776 28250043 PMC5376963

